# The mediating role of internet use in the relationship between working hours and depressive symptoms: an analysis based on cross-lagged models

**DOI:** 10.3389/fpsyg.2025.1656773

**Published:** 2025-09-24

**Authors:** Min Chen, Jie Liu

**Affiliations:** ^1^School of Labor Economics, Capital University of Economics and Business, Beijing, China; ^2^Business College of Beijing Union University, Beijing Union University, Beijing, China

**Keywords:** depressive symptoms, internet use, working hours, mental health, mediation analysis

## Abstract

**Background:**

Amidst the rapid development of the digital economy, prolonged working hours linked to mental health have become a global concern.

**Objective:**

To examine the longitudinal interplay between working hours and depression, and the mediating role of internet use.

**Methods:**

Utilizing a two-wave longitudinal dataset from the China Family Panel Studies (CFPS) spanning 2016 to 2018, we constructed a dynamic analysis sample of 4,900 workers aged 18–70. We employed cross-lagged panel models and semi-longitudinal mediation models to systematically investigate the bidirectional mechanisms between working hours and depressive symptoms. A key focus was to elucidate the mediating pathways of internet use and its heterogeneity across different groups.

**Results:**

The cross-lagged model revealed that working hours, internet use, and depressive symptoms showed dynamic interplay. Working hours, internet use, and depressive symptoms in 2016 positively predicted their respective outcomes in 2018. 2016 working hours positively predicted 2018 internet use (*β* = 0.045, *p* < 0.001) and 2018 depressive symptoms (*β* = 0.027, *p* < 0.05). Crucially, 2016 internet use negatively predicted 2018 depressive symptoms (*β* = −0.032, *p* < 0.01). The semi-longitudinal mediation model demonstrated that internet use mediated the working hours-depression symptoms link (effect = −0.0013, *p* < 0.05), meaning more work led to more internet use, which then reduced depression. This mediation was significant for women, married women, older adults, and stably employed groups. Furthermore, both online entertainment and online social interaction can significantly reduce the risk of depression, but this effect requires a usage frequency of at least 1–2 times per week.

**Conclusion:**

Excessive work harms mental health. Internet use mediates this effect, effectively buffering the psychological harm of overwork.

## 1 Introduction

Work is a foundational aspect of human life, offering avenues for self-actualization, achievement, and material security, while underpinning broader societal progress. However, the equilibrium between work and well-being is increasingly under strain, and long working hours pose substantial risks to mental health. A robust body of evidence links extended work hours to coronary heart disease (Kivimäki et al., 2015), as well as to depression and suicidal tendencies (Lee and Rhie, 2022; Watanabe et al., 2016). These concerns are particularly urgent in East Asia—and especially in China—where recent trends demand closer examination. Economic restructuring and intensifying work demands in China have given rise to extreme work schedules (Gong, 2023), notably the “996” (9 a.m. to 9 p.m., 6 days a week) and “007” (24/7 on availability) models prevalent in the internet and manufacturing sectors. Such schedules have been correlated with significant threats to both physical and mental health.

A substantial body of literature has examined how occupational roles function as stressors impacting workers’ psychological health, and explored behavioral factors such as sleep quality, physical exercise, and family support as potential buffers against this stress (Afonso et al., 2017; Bernstein and McNally, 2018; Qiu et al., 2022; Yin et al., 2023). Yet, with the rapid advancement of information technology, internet use has evolved into an indispensable means for accessing information, engaging in communication, and participating in societal life. Some studies suggest that the proliferation of the internet broadens individuals’ channels for social support and access to information, enabling them to seek broader emotional support and resources when under stress (Ma et al., 2025). Consequently, it is both timely and important to investigate whether internet use can mitigate psychological stress induced by excessive work.

On one hand, the internet provides a platform for social interaction and emotional support, which may buffer the detrimental effects of work-related stress (Qi et al., 2024). On the other hand, excessive screen time and sedentary behavior have been associated with an increased risk of depression (Madhav et al., 2017), and overreliance on virtual environments may undermine real-world emotional connections, fostering loneliness and psychological distress. In light of its dual role as both a coping resource and a potential stressor, internet use represents a dynamic mediator, worthy of rigorous examination in understanding the relationship between working hours and depressive symptoms.

Although internet use plays a growing role in people’s lives, existing research has primarily focused on the direct effects of working hours on depression, without adequately investigating whether internet use can function as an effective means to alleviate work-induced psychological illness. This study aims to address this gap by examining the bidirectional relationship between working hours and depressive symptoms, with particular emphasis on the mediating role of internet use. Employing a two-year cross-lagged panel design, we seek to elucidate the temporal and spatial dynamics of these relationships and to explore potential heterogeneity across different subgroups.

## 2 Literature review

Depression is a common mental disorder characterized by persistent low mood or a loss of pleasure and interest in activities. The Diagnostic and Statistical Manual of Mental Disorders, Fifth Edition (DSM-5) defines depression as a syndrome based on standardized diagnostic criteria, with core features including sustained depressed mood, diminished interest or pleasure (anhedonia), and impairments across multiple domains such as sad, empty, or irritable mood, accompanied by related changes that significantly affect the individual’s capacity to function. Depressive symptoms refer to varying levels of emotional, somatic, and cognitive problems that can be assessed in the general population, making them particularly useful in epidemiological and social science research for identifying risk factors and underlying mechanisms (Liu et al., 2024).

In China, the prevalence of depressive symptoms across the life course—from pre-adolescence to old age—ranges between 20.0 and 41.4% (Pan et al., 2024). Factors influencing depressive symptoms are multidimensional, encompassing not only individual demographic characteristics but also work conditions, income, social capital, and social status (Businelle et al., 2014). With the widespread prevalence of overtime work, the consequences of overwork for depressive symptoms have drawn increasing scholarly attention. However, there is no universally accepted definition of overwork. Due to the availability and objectivity of data, working hours have become the most commonly used indicator in empirical studies (Nie et al., 2015; Kuroda and Yamamoto, 2019).

The relationship between working hours and health outcomes remains contested. Empirical evidence from large-scale cohort studies indicates that long working hours may worsen workers’ mental health. Extended working hours have been shown to elevate the risk of depression and anxiety through mechanisms such as sleep deprivation, deterioration of health behaviors, and reduced time for social interactions and leisure (Virtanen et al., 2011; Kaori et al., 2020). However, other studies suggest that once individual heterogeneity—such as baseline health status and self-selection into jobs—is controlled for, the negative association between working hours and health is attenuated to non-significance (Nie et al., 2015).

With the widespread adoption of information technologies and the increasing prevalence of internet use, scholars have increasingly turned their attention to its association with mental health, although empirical findings remain mixed. On the one hand, internet use may yield positive effects. For example, Chen and Schulz (2016) found that older adults who used the internet for social interaction, learning, entertainment, and consumption could expand their social networks, buffer the loss of health, economic, and social resources associated with aging, and mitigate the impact of adverse life events on depressive symptoms. Similarly, Lam et al. (2020) reported that internet use improved life satisfaction but found no significant association with depressive symptoms. On the other hand, internet use exhibits a dual nature. Mu et al. (2023) suggested that moderate use reduces depressive symptoms among older adults, whereas prolonged use may increase depression risk. In the Chinese context, findings vary substantially across populations and regions. For instance, Wu et al. (2022) found that among adolescents, longer online time was associated with a higher likelihood of depressive symptoms. In contrast, Liu et al. (2024) reported that internet use was negatively associated with depressive symptoms among older adults, with physical activity frequency mediating this relationship. Yang and Wang (2025) found that internet use exacerbated depressive symptoms among rural-to-urban migrants. Additionally, Zhong and Wang (2023) demonstrated that middle-aged and older adults living in provinces with higher levels of internet development reported fewer depressive symptoms compared to those in less developed regions. They further observed that as internet development increases, the mental health benefits of internet use appear to be stronger for women than for men.

In summary, although extensive research has examined the relationship between long working hours and mental health, and evidence has also accumulated regarding the link between internet use and depression, the interactive mechanisms among these three factors have not yet been systematically investigated. In particular, whether and how internet use mediates the association between working hours and depressive symptoms remains underexplored in empirical studies based on longitudinal data and rigorous methods.

## 3 Theoretical framework and research hypotheses

The stress accumulation theory posits that chronic stress arises from relatively persistent conflicts and threats encountered in daily life (Pearlin, 1989). Prolonged exposure to stressors, such as extended working hours, leads to the gradual buildup of psychological strain, ultimately contributing to mental disorders (Haight et al., 2023). When individuals are unable to cope effectively, sustained stress may further result in emotional exhaustion, depersonalization, and diminished personal accomplishment (Maslach et al., 2001). From this perspective, excessive working hours generate enduring stress that increases the likelihood of burnout, role conflict, and eventually depression (Kaori et al., 2020). The theory highlights that the adverse effects of long working hours are not immediate but accumulate over time, underscoring the importance of considering the longitudinal dimensions of work-related stress. Supporting evidence suggests that overtime work elevates the risk of depression through mechanisms such as job burnout, social role conflict, and depletion of emotional resources (Choi et al., 2021).

The social support buffering theory offers a complementary view, suggesting that social support—including emotional, instrumental, and informational support—can mitigate the detrimental effects of stress (Alloway and Bebbington, 1987; Cantor et al., 1995; Qi et al., 2024). The internet has introduced novel forms of communication, shifting interaction from traditional letters and telephone calls toward email, video conferencing, online forums, and instant messaging. These technologies enable the continuous and immediate exchange of information, greatly expanding both the scope and frequency of social interactions. As such, the internet has become a crucial avenue for broadening social support networks (Nabi et al., 2013). This function is particularly salient when face-to-face interactions are constrained by occupational demands, as online platforms allow sustained connection and emotional exchange.

**Figure 1:**
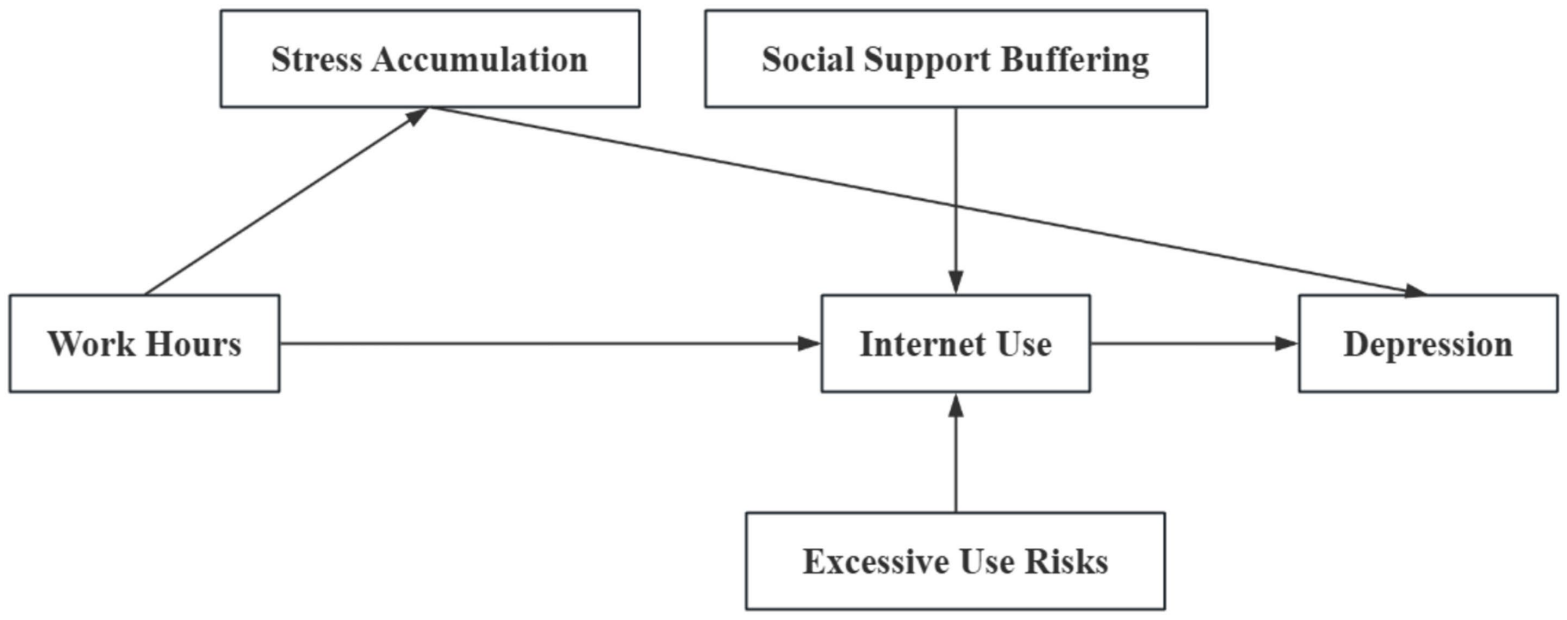
Theoretical framework analysis.

Nevertheless, while online support can buffer certain stressors, it also carries risks. Excessive internet use may precipitate psychological distress, particularly when it supplants offline relationships or promotes passive media consumption, thereby intensifying feelings of isolation or inadequacy (Nie, 2001; Madhav et al., 2017). Moreover, the impact of internet use on depressive symptoms may vary according to individual characteristics. For instance, factors such as gender, age, and marital status can modify the actual effect of internet use on depression (Wu et al., 2022; Liu et al., 2024; Yang and Wang, 2025; Wei et al., 2023). Thus, internet use can function both as a source of support and as a potential stressor, making it a complex mediator in the relationship between work and depression. Figure 1 presents the theoretical framework of working hours, Internet use, and depressive symptoms based on the Stress Accumulation Theory and the Social Support Buffering Theory.

Although various mediating factors can be considered in this relationship, internet use is distinctive due to its dual nature as both a stress-relieving resource and a potential stress inducer. The stress accumulation theory and the social support buffering theory together provide complementary frameworks for understanding how internet use may shape the impact of working hours on depressive symptoms. On one hand, internet use can serve as a coping mechanism by offering workers access to emotional support; on the other hand, overuse may introduce new stressors and exacerbate depressive outcomes. By integrating these theoretical perspectives, the following hypotheses are proposed:

*H*1: There is a positive relationship between working hours and depressive symptoms, such that longer working hours significantly increase the level of depressive symptoms.

*H*2: Internet use mediates the relationship between working hours and depressive symptoms.

*H*3: The mediating effect of internet use varies across different population subgroups.

## 4 Research subjects and design

### 4.1 Research subjects

#### 4.1.1 Research data

The data for this study come from the China Family Panel Studies (CFPS), a nationally representative longitudinal survey funded by Peking University and the National Natural Science Foundation of China (Li et al., 2025b). Initiated in 2010 and conducted biennially, the CFPS uses computer-assisted interviews to collect extensive information from all household members. Publicly available data cover the years 2010, 2012, 2014, 2016, 2018, and 2020. Because depression-related questions were absent in 2010 and 2014, inconsistent in 2012, and internet-use criteria changed in 2020, we restricted our analysis to the 2016 and 2018 waves to ensure comparability. The 2016 wave included 36,892 respondents, and the 2018 wave included 37,354. The 2018 sample comprised all individuals successfully interviewed in 2016, along with participants surveyed between 2010 and 2014 who had not been tracked in 2016. We only used secondary data from the CFPS database which is open for the public. So we did not collect data from the respondents and thus exempted by the Institutional Review Board (IRB) of our university.

**Figure 2:**
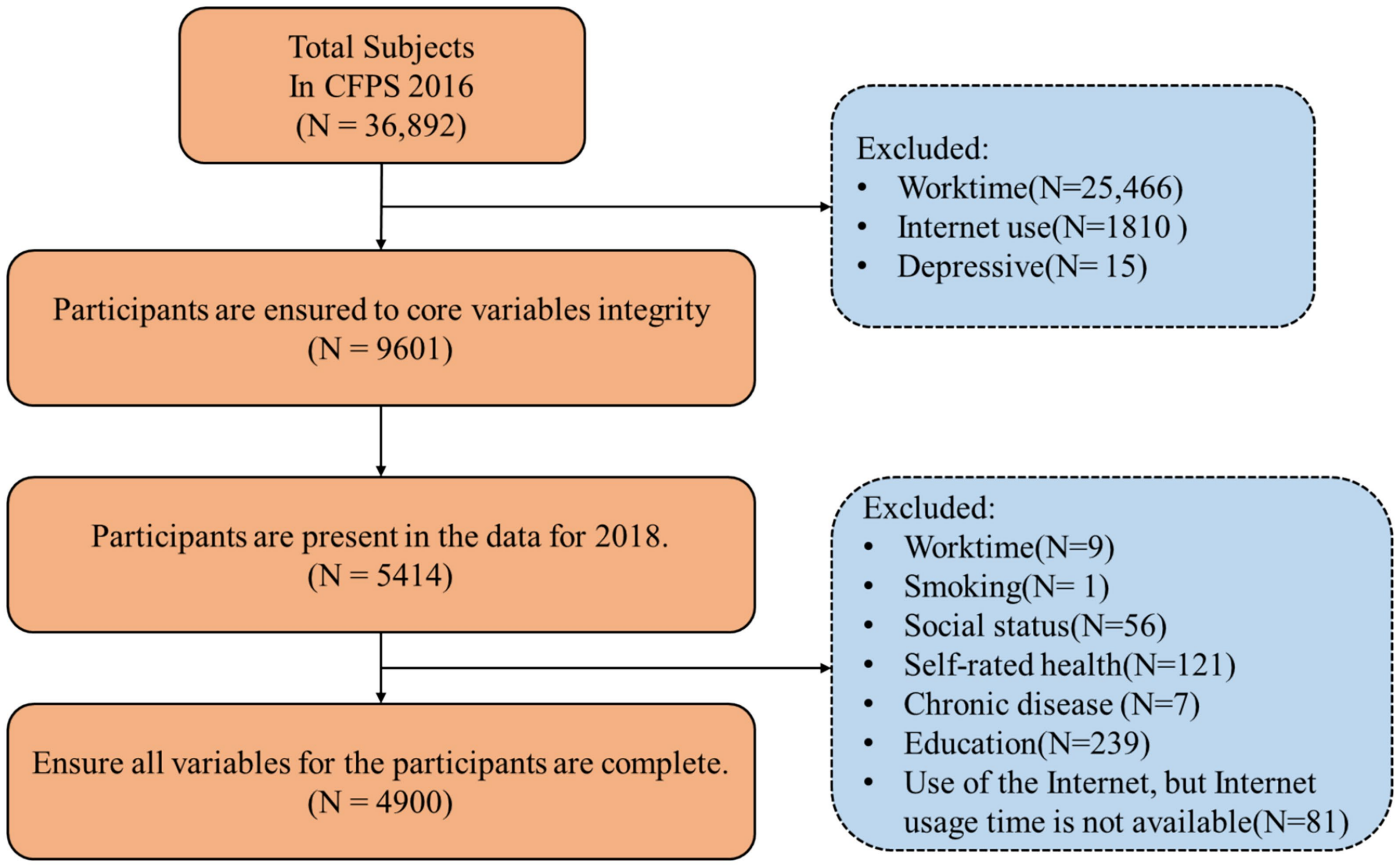
Sample selection process.

We began with 9,601 individuals in 2016, excluding those missing key variables such as working hours, internet use, or depressive symptoms. Matching with the 2018 dataset yielded 5,414 individuals with complete information for both years. After further excluding cases with missing control variables or internet usage data, the final sample comprised a balanced two-wave panel of 4,900 participants (2,754 men and 2,146 women), all observed in both 2016 and 2018. Each wave thus contains data from the same 4,900 individuals. The sample selection process is illustrated in Figure 2.

#### 4.1.2 Survey methodology

The China Family Panel Studies (CFPS) employs the Computer-Assisted Personal Interview (CAPI) method, marking its first large-scale application in a longitudinal survey in China. Fieldwork is organized by the China Social Science Survey Center at Peking University, which recruits and trains interviewers. Before household visits, interviewers contact local village or community leaders and present official letters of introduction to gain support. During interviews, standardized procedures are followed to complete questionnaires online, with real-time technical assistance available in cases of system or network issues. Uploaded data are jointly reviewed by the data and quality control teams to ensure accuracy and reliability. The baseline survey covered 25 provinces/municipalities/autonomous regions, 162 districts/counties, and 649 villages/communities, yielding 19,986 effective household samples. The survey includes household, adult, child, and community questionnaires, offering a comprehensive view of the micro-level realities of Chinese family life.

### 4.2 Variable selection and research design

#### 4.2.1 Working hours

We measured working hours using the CFPS questionnaire item: “In the past 12 months, how many hours do you usually work per week in your current job? (Exclude lunch breaks but include overtime, regardless of compensation).” This measure captures both regular hours and overtime, thereby providing a more accurate reflection of an individual’s labor market engagement.

#### 4.2.2 Internet use

Internet use was assessed with two survey questions: “Do you access the internet via mobile devices (including smartphones and tablets)?” and “Do you use a computer to access the internet?” Participants who answered “yes” to either question were classified as internet users (Internet use = 1); otherwise, they were considered non-users. Among internet users, individuals were further classified based on their leisure internet usage time: those in the bottom 50% were categorized as “low internet use,” while those in the top 50% (including the median) were classified as “high internet use.” To further examine the mediating role of different types of internet use, we employed the frequency of online entertainment (e.g., watching videos, downloading music) and online social interaction (e.g., chatting, posting on Weibo) as mediating variables. Response options included “almost daily,” “3–4 times per week,” “1–2 times per week,” “2–3 times per month,” “once per month,” “every few months,” and “never.” For the odds ratios analysis, participants who reported engaging in online entertainment or social use almost daily, 3–4 times per week, or 1–2 times per week were categorized as high internet use, while those reporting 2–3 times per month, once per month, or every few months were classified as low internet use.

#### 4.2.3 Depressive symptoms

The CFPS uses the Center for Epidemiological Studies Depression Scale (CES-D) to measure depressive symptoms. The CES-D, a widely recognized self-report scale, assesses depressive symptoms in both clinical and non-clinical settings and is known for its reliability and validity (Li et al., 2025b; Tan et al., 2023). Originally comprising 20 items, the CES-D was streamlined to eight core items for consistency between the 2016 and 2018 datasets, following Liu et al. (2024). These items are: “I felt depressed,” “I felt everything I did was an effort,” “My sleep was restless,” “I felt happy,” “I felt lonely,” “I enjoyed life,” “I felt sad,” and “I could not get going.” Respondents rate these feelings or behaviors on a scale from 0 (rarely or none of the time) to 3 (most or all of the time). The items “I felt happy” and “I enjoyed life” are reverse-scored to maintain the logical consistency of the scale. The CES-D8 total score ranges from 0 to 24, with scores ≥9 indicating clinically significant depressive symptoms (Wu et al., 2023). The scale demonstrated good reliability, with Cronbach’s α coefficients of 0.869 and 0.831 in 2016 and 2018, respectively.

#### 4.2.4 Control variables

To account for potential confounding factors, we controlled for demographic, socioeconomic, health, and lifestyle variables (Choi et al., 2021; Kong et al., 2023). Demographic variables included age, gender (male = 1, female = 0), years of education, and marital status (married = 1, unmarried = 0). Socioeconomic status was measured by self-rated social standing (1–5, with higher scores indicating higher status). Health variables comprised self-reported chronic illness diagnosis within the past 6 months (yes = 1, no = 0) and self-rated health (1–5, with higher scores indicating better health). Lifestyle variables included smoking (yes = 1, no = 0) and alcohol consumption (yes = 1, no = 0).

#### 4.2.5 Statistical analysis

To comprehensively characterize the sample, statistical analyses of participants’ demographic and personal variables were conducted. Data were managed and analyzed using IBM SPSS 26.0, including the calculation of means, standard deviations, and frequency distributions for each variable, while categorical variables are expressed as n (%). A Chi-square test was employed for group comparisons, and Pearson correlation analysis was conducted to examine relationships among key variables, allowing for a preliminary identification of potential associations between working hours, internet use, and depressive symptoms.

Subsequently, a cross-lagged panel model with mediation will be employed to explore the complex longitudinal relationships among working hours, internet use, and depressive symptoms. The specific model, see Equations (1) to (3):


(1) $$X_{t+1}=\beta_{X}X_{t}+\varepsilon_{X(t+1)}\;$$

(2) $$M_{t+1}=\beta_{M}M_{t}+\beta_{a}X_{t}+\varepsilon_{M(t+1)}\;$$

(3) $$Y_{t+1}=\beta_{Y}Y_{t}+\beta_{b}M_{t}+\beta_{c}X_{t}+\varepsilon_{Y(t+1)}\;$$

In this model, X represents the independent variable, M serves as the mediating variable, and Y is the dependent variable. Each variable in the model is dependent on both its own prior measurement level and the prior measurement levels of other variables. The regression results of each variable on the prior measurement levels of other variables are referred to as lagged effects ( $\beta_{a},\beta_{b},\beta_{c}$ represented the lagged regression coefficients in the model). The regression results of each variable on its own prior measurement level are known as autoregressive effects ( $\beta_{X},\beta_{M},\beta_{Y}$ represented the autoregressive coefficients in the model). The cross-lagged model aims to clarify the dynamic interactions between variables, emphasizing causal paths in time series, and can effectively capture the bidirectional interactive relationships between variables (Liu et al., 2024).

Furthermore, to investigate whether internet use mediates the longitudinal association between working hours and depressive symptoms, we employed a semi-longitudinal mediation model. The semi-longitudinal mediation model proposed by Cole and Maxwell (2003) makes it possible to examine mediation relationships using two waves of data. The advantage of this model lies in its ability to effectively capture mediating effects within a time series, especially with two-wave longitudinal data. It can better capture dynamic changes between variables and offers greater accuracy than cross-sectional mediation analysis. For the specific model, see Equations (4) and (5):


(4) $$M_{t+1}=\beta_{M}M_{t}+{\mathrm{aX}}_{t}+\varepsilon_{M(t+1)}\;$$

(5) $$Y_{t+1}=\beta_{Y}Y_{t}+{\mathrm{bM}}_{t}+\varepsilon_{Y(t+1)}\;$$

**Figure 3:**
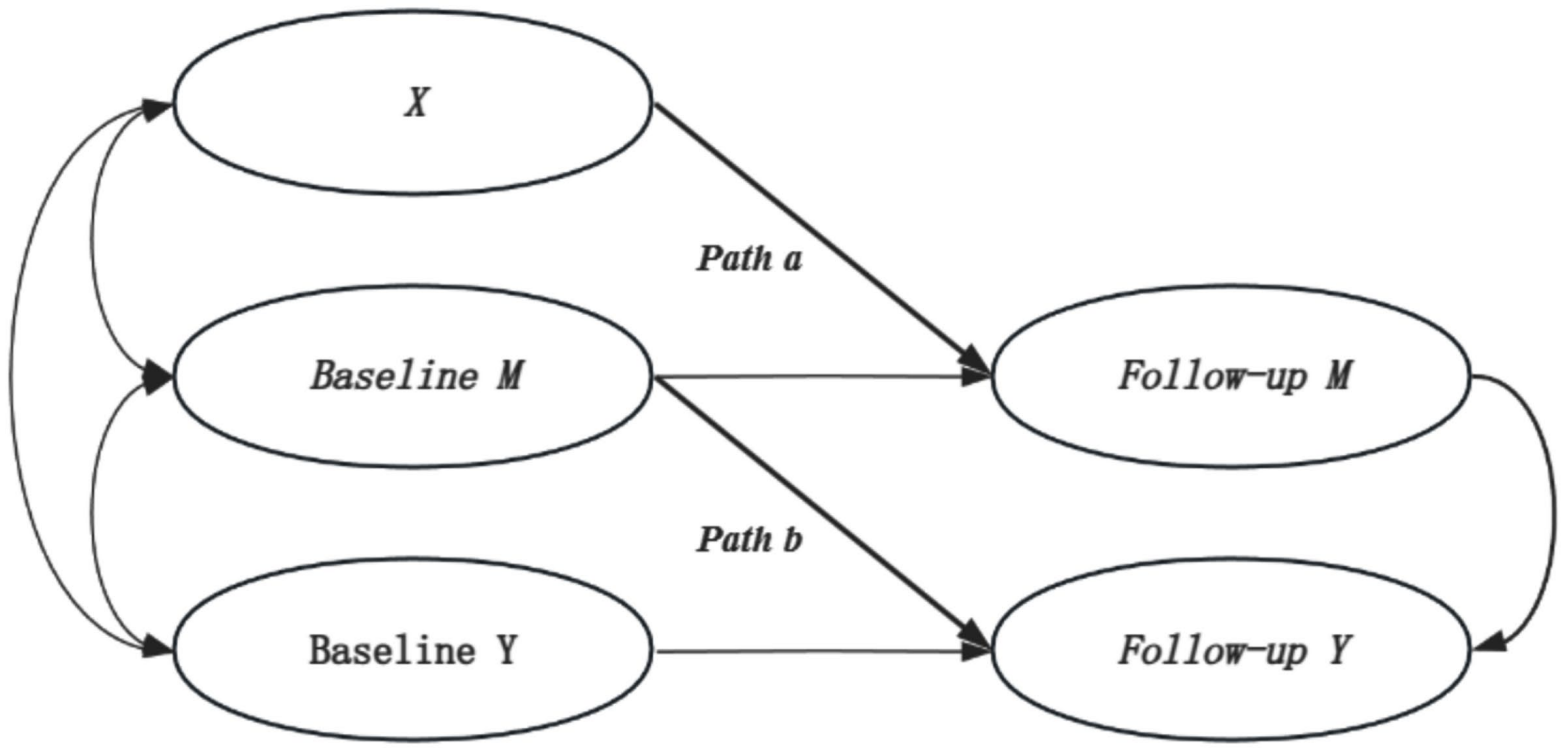
Semi-longitudinal mediation model.

The semi-longitudinal mediation model primarily focuses on two key longitudinal tests: (1) Path a, which represents the regression coefficient of the baseline independent variable (X) on the follow-up mediator (M); (2) Path b, which signifies the estimated influence of the baseline mediator (M) on the follow-up dependent variable (Y). The mediation effect of M is considered significant if both Path a and Path b are statistically significant. It’s important to note that, unlike the more comprehensive cross-lagged model, the semi-longitudinal mediation model adopts a more streamlined approach. Its core focus is on a specific mediating pathway (independent variable → mediator → dependent variable), rather than examining the dynamic causal relationships among all variables. This focused strategy helps validate the mediating role of a particular mechanism, offering more precise evidence to clarify how internet use potentially mediates the relationship between working hours and depressive symptoms. Both the cross-lagged and semi-longitudinal mediation models were estimated using Mplus 8.0, with a schematic representation provided in Figure 3.

Finally, Stata 16.0 was used to estimate the odds ratios (ORs) and 95% confidence intervals (CIs) for depressive symptoms across different levels of internet use. Individuals reporting no internet use served as the reference group. A binary panel Logit regression model was employed to estimate the ORs and 95% CIs for the development of depressive symptoms at varying levels of internet use. This model was based on two waves of longitudinal data (2016 and 2018), allowing for the effective control of individual fixed effects. By accounting for these fixed effects, this approach reduces the influence of potential confounding variables, thereby providing a more accurate estimation of the relationship between internet use and depressive symptoms.

## 5 Results

### 5.1 Descriptive statistics and correlations

Table 1 presents the demographic characteristics of participants at T1 (2016). Among the 4,900 participants, ages ranged from 18 to 70 years. Overall, 43.8% were male, and 77.35% were married. At T1, the average working hours were 48.55 h per week, with approximately 74.78% of participants exceeding the statutory working hours. Over half of the participants (59.84%) reported using the internet. Among these internet users, the sample was further divided into groups based on quartiles of weekly leisure internet use to examine the distribution of different usage levels. The mean score for depressive symptoms among workers was 5.11. By T2 (2018), the average working hours remained similar at 48.54 h per week, with 62.69% of participants exceeding the statutory working hours, while internet usage increased to 66.63%. The mean depressive symptom score rose slightly to 5.45. Statistically significant differences in the distribution of depressive symptoms were observed across various demographic, socioeconomic, health, lifestyle, and internet usage factors (*p* < 0.05), see Table 1.

**Table 1:** Statistical characteristics of T1 participants.

Variable	Total (*n* = 4,900)	*p*
Demographic Factors		
Age(years)		**<0.001**
18–34	2,192 (44.73%)	
35–44	973 (19.86%)	
>44	1735 (35.41%)	
Gender, n(%)		**<0.001**
Male	2,146 (43.80%)	
Female	2,754 (56.2%)	
Marital status, n(%)		**<0.001**
No spouse	1,110 (22.65%)	
Spouse	3,790 (77.35%)	
Education		**<0.001**
Illiterate	791 (16.14%)	
Elementary school	999 (20.39%)	
Junior high school	1,490 (30.41%)	
High school	821 (16.76%)	
College	431 (8.80%)	
University	333 (6.80%)	
Master	33 (0.67%)	
Doctor	2 (0.04%)	
Socioeconomic Status Factors		
Self-rated social status, Mean (SD)	2.67 (1.02)	**<0.001**
Health factors		
Self-rated health, Mean (SD)	2.82 (1.69)	**<0.001**
Chronic Diseases, n (%)		**<0.001**
Yes	4,343 (88.63%)	
No	557 (11.37%)	
Lifestyle Factors		
Smoking, n (%)		**<0.001**
Yes	1,661 (33.90%)	
No	3,239 (66.10%)	
Drinking, n (%)		**<0.001**
Yes	781 (15.94%)	
No	4,119 (84.06%)	
Main Variables		
Worktime T1, Mean (SD)	48.55 (22.17)	
≤ 40 h	1,236 (25.22%)	
41 ~ 55 h	970 (19.80%)	
>55 h	2,694 (54.98%)	
Worktime T2, Mean (SD)	48.54 (22.62)	
≤ 40 h	1828 (37.31%)	
41 ~ 55 h	978 (19.96%)	
>55 h	2094 (42.73%)	
Internet use T1, n (%)	2,932 (59.84%)	**<0.001**
Internet use T2, n (%)	3,265 (66.63%)	**<0.001**
Online Time T1, n(%)		
5 h	789 (26.91%)	
10 h	762 (25.99%)	
20 h	729 (24.86%)	
84 h	652 (22.24%)	
Online Time T2, n(%)		
6 h	855 (26.19%)	
14 h	1,279 (39.17%)	
20 h	358 (10.96%)	
90 h	773 (23.68%)	
Depression symptoms T1, Mean (SD)	5.11 (3.78)	
Depression symptoms T2, Mean (SD)	5.45 (3.72)	

T1 to T2 refers to the period from 2016 to 2018; SD stands for standard deviation, < 0.001 indicates that the p-value for the statistical test is less than 0.001.

Table 2 presents the Pearson correlation coefficients between working hours, internet use, and depression in the first and second waves of the cohort. The results show a significant positive correlation between working hours and internet use. Depression symptoms are significantly positively correlated with working hours and significantly negatively correlated with internet use.

**Table 2:** Pearson correlation coefficients.

	1	2	3	4	5	6
1.worktime T1	1					
2.worktime T2	0.400^**^	1				
3. Internet use T1	0.129^**^	0.152^**^	1			
4. Internet use T2	0.125^**^	0.138^**^	0.650^**^	1		
5. Depression symptoms T1	0.033^*^	0.034^*^	−0.054^**^	−0.043^**^	1	
6. Depression symptoms T2	0.035^*^	0.042^**^	−0.054^**^	−0.052^**^	0.464^**^	1

*p < 0.05, **p < 0.01, ***p < 0.001.

### 5.2 Cross-lagged model

To examine the causal relationships among working hours, internet use, and depressive symptoms, a cross-lagged panel model was constructed, controlling for age, gender, marital status, years of education, self-rated social status, self-rated health, chronic diseases, smoking, and drinking. The model fit was evaluated using multiple indices, including the Comparative Fit Index (CFI), Standardized Root Mean Square Residual (SRMR), Root Mean Square Error of Approximation (RMSEA), and Tucker–Lewis Index (TLI), all of which indicated a good fit. Although the chi-square test was not statistically significant—likely due to the large sample size—consideration of the other fit indices suggests that the overall model fit was acceptable (CFI = 0.991, SRMR = 0.016, RMSEA = 0.048, TLI = 0.956, $\chi^{2}/\mathrm{df}$ = 12.091).

**Figure 4:**
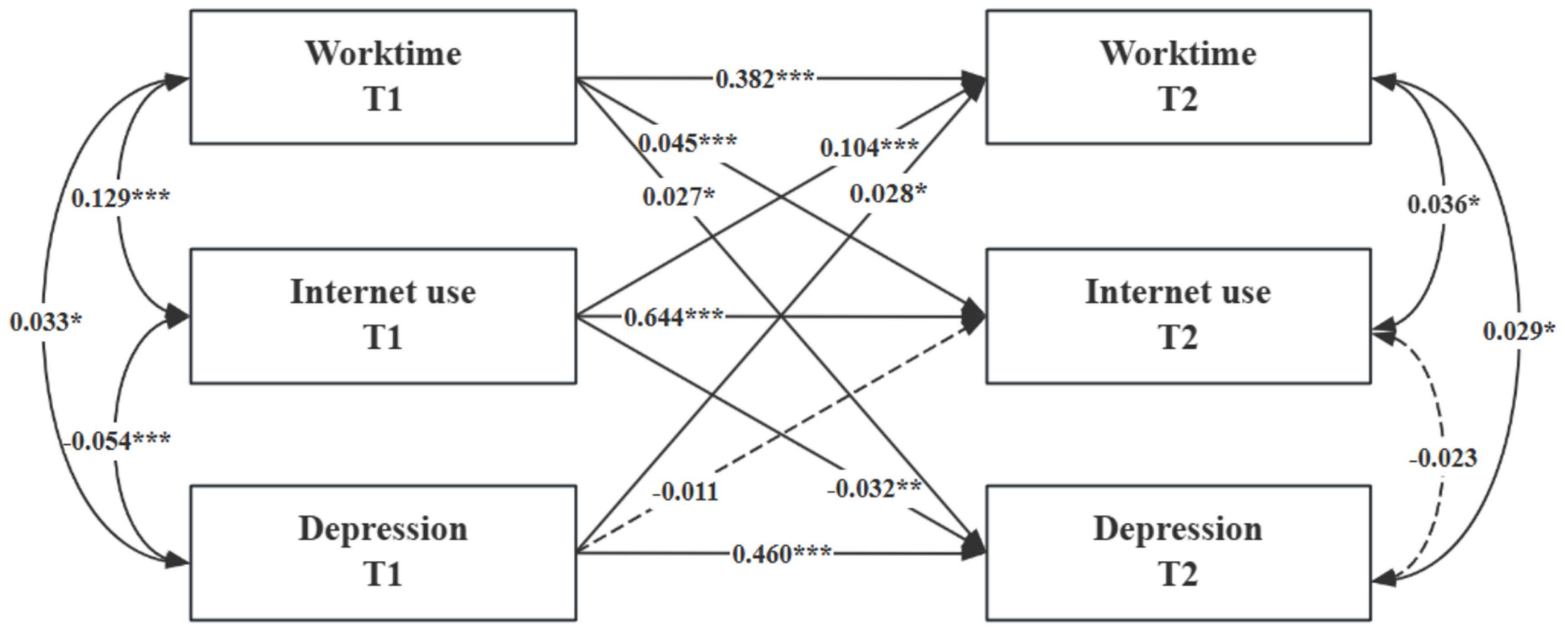
Cross-lagged regression of working hours, internet usage, and depression. *p < 0.05, **p < 0.01, ***p < 0.001; T1, Time 1; T2, Time 2; Covariates, residuals, and residual correlations are not listed for graphical simplicity. The figure is the standardized regression coefficient; the solid line indicates that the regression coefficient is significant, and the dotted line indicates that the regression coefficient is not significant.

In terms of autoregressive effects, T1 working hours, internet use, and depressive symptoms each positively predicted their respective T2 counterparts. Regarding lagged effects, T1 working hours positively predicted T2 internet use ( β = 0.045, *p* < 0.001), and T1 working hours positively predicted T2 depressive symptoms ( β = 0.027, *p* < 0.05). Conversely, T1 internet use negatively predicted T2 depressive symptoms ( β = − 0.032, *p* < 0.01). For reverse pathways, T1 internet use also positively predicted T2 working hours, and T1 depressive symptoms positively predicted T2 working hours; however, the effect of T1 depressive symptoms on T2 internet use was not statistically significant. These findings highlight a dynamic interplay among working hours, internet use, and depressive symptoms, illustrating both the aggravating role of working hours on depressive symptoms and the mitigating effect of internet use. See Figure 4.

### 5.3 Mediation effect analysis

To examine the potential mechanisms linking working hours, internet use, and depressive symptoms, a semi-longitudinal mediation analysis was conducted based on the methodological framework proposed by Cole and Maxwell (2003). This semi-longitudinal mediation model offers advantages in capturing causal relationships within temporal sequences, allowing for a more accurate identification of dynamic changes between variables in two-wave panel data compared with traditional cross-sectional mediation analyses.

The analysis focused on two key pathway coefficients: (*a*) the effect of T1 working hours on T2 internet use (Path *a*), and (*b*) the effect of T1 internet use on T2 depressive symptoms (Path *b*). T1 working hours positively predicted T2 internet use (*a* = 0.045, *p* < 0.001), whereas T1 internet use negatively predicted T2 depressive symptoms (*b* = −0.029, *p* < 0.01). These results indicate that longer working hours increase internet use, which in turn effectively reduces the likelihood of depressive symptoms. While both Path *a* and Path *b* were statistically significant, a Sobel test was conducted to verify the robustness of the semi-longitudinal mediation results (Sobel, 1982). The Sobel test yielded a Z-value of −2.365, which was statistically significant (*p* < 0.05), with a mediation effect of −0.0013 ( a∗b ). These findings support the mediating role of internet use in the relationship between working hours and depressive symptoms.

**Figure 5:**
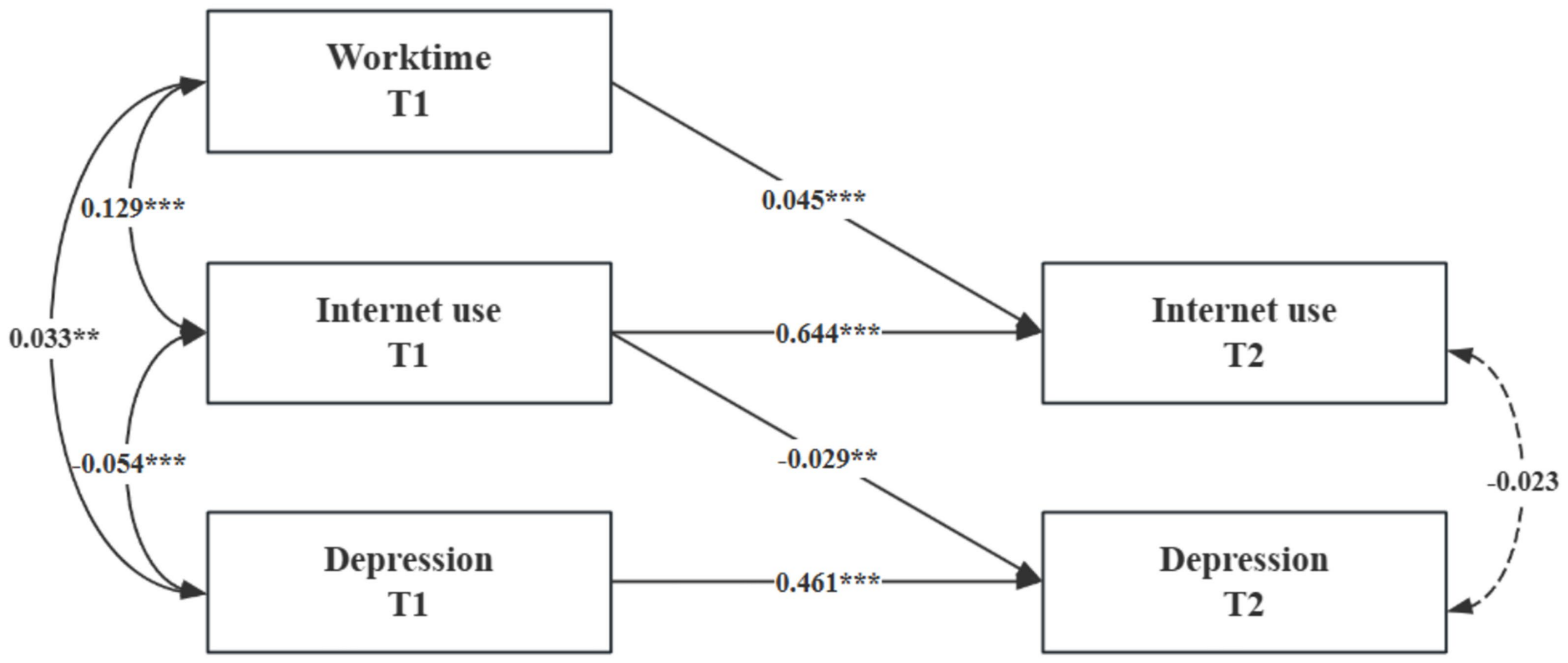
Semi-longitudinal mediation analysis of working hours, internet use, and depressive symptoms. *p < 0.05, **p < 0.01, ***p < 0.001; Covariates, residuals, and residual correlations are not listed for graphical simplicity. The figure is the standardized regression coefficient; the solid line indicates that the regression coefficient is significant, and the dotted line indicates that the regression coefficient is not significant.

Although the mediation effect size is relatively modest, the significant result of the Sobel test provides strong evidence that internet use mediates the relationship between working hours and depressive symptoms. Given the complex etiology of depression, even a small mediation effect can have meaningful implications for prevention and intervention strategies. This mediating effect highlights the potential of internet use in alleviating depressive symptoms, and long-term interventions may yield cumulative benefits at the individual level. From a public health perspective, even modest improvements can translate into substantial population-level benefits. See Figure 5.

### 5.4 Heterogeneity analysis

Table 3 presents results of the semi-longitudinal mediation model across subgroups, including Path *a* (effect of T1 working hours [T1WT] on T2 internet use [T2IU]) and Path *b* (effect of T1 internet use [T1IU] on T2 depression [T2DE]). Age groups were categorized as young (18–34 years), middle-aged (35–44 years), and older workers (≥ 45 years). While the United Nations defines youth as 16–24 years, we extended the upper age limit for young workers to 34 years to account for delayed labor market entry due to educational expansion in post-reform China (Huang et al., 2022). Drawing on prior research (Baek et al., 2024), employment stability was measured using the contractual status reported in surveys. Self-employed individuals and those with inconsistent labor contract status between 2016 and 2018 were excluded to ensure sample consistency, retaining only participants with stable contractual status across both waves for longitudinal validity (see Table 3).

**Table 3:** Heterogeneity analysis.

Subgroups	*a*(T1WT → T2IU)	*b*(T1IU → T2DE)	Sobel(*p*-value)	Test
Man	0.025	0.002	/	/
Woman	0.068**	−0.068**	0.006	−2.738
Married	0.119***	−0.043**	0.007	−2.675
Unmarried	0.084	−0.043	/	/
Married women	0.132***	−0.526***	0.0002	−3.708
Unmarried women	0.026	−0.045*	/	/
Youth	−0.009	−0.043	/	/
Middle-Aged	0.290	−0.035	/	/
Older	0.124*	−0.052**	0.064	−1.852
Employment Instability	0.032	0.016	/	/
Employment Stability	0.066**	−0.061*	0.124	−1.540
Urban	0.018*	−0.020	/	/
Country	0.058**	−0.038*	0.062	−1.870
Entertainment	−0.031	−0.026**	/	/
Social	−0.121	−0.027*	/	/

*p < 0.05, **p < 0.01, ***p < 0.001. T1WT → T2IU represents the regression coefficient of T1 working hours on T2 internet use, T1IU → T2DE denotes the regression coefficient of T1 internet use on T2 depressive symptoms, Sobel (p-value) indicates the p-value from the Sobel test, and Test refers to the Z-value obtained from the Sobel test.

In the gender group, women’s T1 working hours positively predicted T2 internet use (*a* = 0.068, *p* < 0.01), and T1 internet use negatively predicted T2 depressive symptoms (*b* = −0.068, *p* < 0.01). The Sobel test was passed, confirming the mediating role of internet use in the relationship between working hours and depressive symptoms for women, with a mediation effect of −0.004624 ( a∗b ). Among marital status groups, married individuals’ T1 working hours positively predicted T2 internet use (*a* = 0.119, *p* < 0.001), and T1 internet use negatively predicted T2 depressive symptoms (*b* = −0.043, *p* < 0.01). The Sobel test yielded a *p*-value of 0.0074, indicating a mediating effect of internet use in the relationship between working hours and depressive symptoms for married workers.

Given the pronounced mediating effects observed particularly among women and married individuals, further analysis of internet use stratified by marital status within the female demographic may yield more insightful results. Among married women, T1 working hours positively predicted T2 internet use (*a* = 0.132, *p* < 0.001), and T1 internet use negatively predicted T2 depressive symptoms (*b* = −0.526, *p* < 0.001). Sobel’s test confirmed a significant mediation effect (*p* = 0.0002), indicating that internet use mediates the relationship between working hours and depression in this group. In contrast, among unmarried women, T1 working hours did not significantly predict T2 internet use, although T1 internet use remained a negative predictor of T2 depressive symptoms (*b* = −0.045, *p* < 0.05).

In the age-stratified analysis, only the elderly group showed statistically significant path *a* and path *b* (*a* = 0.124, *p* < 0.05; *b* = −0.052, *p* < 0.01), but the Sobel test was not passed. In the employment stability group, stable employment individuals’ T1 working hours positively predicted T2 internet use, and T1 internet use also negatively predicted T2 depressive symptoms (*a* = 0.066, *p* < 0.01; *b* = −0.061, *p* < 0.05). However, the Sobel test was not significant (*p* > 0.05), indicating a lack of statistical significance. Participants were stratified by residential location into urban and rural groups. Among rural individuals, T1 working hours positively predicted T2 internet use, and T1 internet use negatively predicted T2 depressive symptoms; however, the Sobel test did not reach statistical significance. In contrast, no significant effects were observed for Path a or Path b among urban participants.

Additionally, we further examined specific types of internet use, including recreational use (e.g., watching videos, downloading music) and social use (e.g., chatting, posting on social media), to explore how different internet activities may influence the relationship between working hours and depressive symptoms. The results indicated that increased frequency of both recreational and social internet use was associated with a reduction in depressive symptoms. However, longer working hours tended to reduce the likelihood of engaging in either recreational or social internet use, although these estimates were not statistically significant. In conclusion, internet use acts as a mediator in the relationship between working hours and depressive symptoms for women and married individuals, particularly for married women, whereas the mediation paths were also statistically significant for the elderly and those in stable employment. See Table 3.

### 5.5 Odds ratio analysis

Table 4 presents odds ratios (ORs) and 95% confidence intervals (CIs) for depression risk in high (HI) and low (LI) internet use groups, using non-internet users (NI) as the reference. All OR values were below 1, indicating that both HI and LI groups had lower depression risks compared to NI. Notably, differences in depression risk between HI and LI groups did not reach statistical significance in middle-aged, elderly, and unstable employment subgroups. Moreover, longer internet use was associated with a lower likelihood of depressive symptoms among participants residing in both urban and rural areas. When examining internet use by purpose, neither social nor recreational use had a significant effect on depressive symptoms at low usage frequency. However, at higher usage frequencies, the likelihood of depression decreased significantly, with recreational use demonstrating a stronger protective effect than social use (see Table 4).

**Table 4:** Odds ratios of depression across internet use groups.

Subgroup	OR(95%CI)	*p*
Total		
NI	1.00	
HI	0.65 (0.55 ~ 0.78)	**<0.01**
LI	0.77 (0.67 ~ 0.89)	**<0.01**
Male		
NI	1.00	
HI	0.47 (0.36 ~ 0.61)	**<0.01**
LI	0.50 (0.40 ~ 0.62)	**<0.01**
Female		
NI	1.00	
HI	0.55 (0.43 ~ 0.70)	**<0.01**
LI	0.68 (0.56 ~ 0.84)	**<0.01**
Youth		
NI	1.00	
HI	0.57 (0.46 ~ 0.71)	**<0.01**
LI	0.65 (0.54 ~ 0.78)	**<0.01**
Middle		
NI	1.00	
HI	0.98 (0.72 ~ 1.35)	0.419
LI	0.93 (0.74 ~ 1.16)	0.206
Old		
NI	1.00	
HI	0.67 (0.42 ~ 1.06)	0.062
LI	0.81 (0.62 ~ 1.08)	0.505
Unstable employment		
NI	1.00	
HI	0.78 (0.55 ~ 1.10)	0.161
LI	0.80 (0.59 ~ 1.08)	0.147
Stable employment		
NI	1.00	
HI	0.65 (0.47 ~ 0.90)	**<0.01**
LI	0.57 (0.41 ~ 0.79)	**<0.01**
Married		
NI	1.00	
HI	0.29 (0.21 ~ 0.38)	**<0.01**
LI	0.26 (0.20 ~ 0.34)	**<0.01**
Unmarried		
NI	1.00	
HI	0.35 (0.26 ~ 0.49)	**<0.01**
LI	0.33 (0.24 ~ 0.45)	**<0.01**
Married women		
NI	1.00	
HI	0.15 (0.09 ~ 0.28)	**<0.01**
LI	0.15 (0.08 ~ 0.25)	**<0.01**
Unmarried women		
NI	1.00	
HI	0.56 (0.41 ~ 0.78)	**<0.01**
LI	0.72 (0.57 ~ 0.91)	**<0.01**
Urban		
NI	1.00	
HI	0.62 (0.43 ~ 0.91)	**<0.05**
LI	0.71 (0.51 ~ 0.97)	**<0.05**
Country		
NI	1.00	
HI	0.42 (0.29 ~ 0.63)	**<0.01**
LI	0.58 (0.43 ~ 0.79)	**<0.01**
Entertainment		
NI	1.0	
HI	0.49 (0.41 ~ 0.61)	**<0.01**
LI	0.70 (0.44 ~ 1.09)	0.115
Social		
NI	1.00	
HI	0.51 (0.42 ~ 0.62)	**<0.01**
LI	0.84 (0.48 ~ 1.44)	0.527

NI represents the non-internet users group (control group), HI represents high internet users, and LI represents low internet users. “< 0.05” and “< 0.01” denote that the *p*-values for the statistical tests are less than 0.05 and 0.01, respectively.

Additionally, a robustness check was conducted using a depressive symptom score ≥ 8 as the outcome. The results were largely consistent with those presented in Table 4, further validating the previous findings, see Table 5.

**Table 5:** Odds ratios of depression across internet use groups (thresholds≥8).

Subgroup	OR(95%CI)	*p*
Total		
NI	1	
HI	0.60 (0.47 ~ 0.74)	**<0.01**
LI	0.75 (0.63 ~ 0.89)	**<0.01**
Male		
NI	1	
HI	0.59 (0.48 ~ 0.74)	**<0.01**
LI	0.75 (0.62 ~ 0.89)	**<0.01**
Female		
NI	1	
HI	0.44 (0.32 ~ 0.61)	**<0.01**
LI	0.66 (0.51 ~ 0.85)	**<0.01**
Youth		
NI	1	
HI	0.54 (0.35 ~ 0.83)	**<0.01**
LI	0.71 (0.59 ~ 0.81)	**<0.01**
Middle		
NI	1	
HI	0.89 (0.54 ~ 1.46)	0.419
LI	0.85 (0.59 ~ 1.21)	0.357
Old		
NI	1	
HI	0.78 (0.57 ~ 1.06)	0.110
LI	0.85 (0.67 ~ 1.08)	0.193
Unstable employment		
NI	1	
HI	0.94 (0.59 ~ 1.51)	0.796
LI	1.01 (0.68 ~ 1.49)	0.969
Stable employment		
NI	1	
HI	0.60 (0.47 ~ 0.74)	**<0.01**
LI	0.75 (0.63 ~ 0.89)	**<0.01**
Married		
NI	1	
HI	0.21 (0.13 ~ 0.36)	**<0.01**
LI	0.24 (0.14 ~ 0.41)	**<0.01**
Unmarried		
NI	1	
HI	0.66 (0.51 ~ 0.85)	**<0.01**
LI	0.85 (0.71 ~ 1.04)	0.11
Married women		
NI	1	
HI	0.11 (0.04 ~ 0.29)	**<0.01**
LI	0.18 (0.07 ~ 0.44)	**<0.01**
Unmarried women		
NI	1	
HI	0.53 (0.37 ~ 0.76)	**<0.01**
LI	0.75 (0.58 ~ 0.98)	**<0.01**
Urban		
NI	1	
HI	0.66 (0.48 ~ 0.91)	**<0.05**
LI	0.82 (0.63 ~ 1.06)	0.135
Country		
NI	1	
HI	0.62 (0.45 ~ 0.87)	**<0.01**
LI	0.77 (0.60 ~ 0.99)	**<0.01**
Entertainment		
NI	1	
HI	0.64 (0.54 ~ 0.77)	**<0.01**
LI	0.83 (0.57 ~ 1.20)	0.316
Social		
NI	1	
HI	0.67 (0.56 ~ 0.79)	**<0.01**
LI	0.78 (0.48 ~ 1.26)	0.312

NI represents the non-internet users group (control group), HI represents high internet users, and LI represents low internet users. “< 0.05” and “< 0.01” denote that the p-values for the statistical tests are less than 0.05 and 0.01, respectively.

## 6 Discussion

This study analyzed the bidirectional causal relationships between working hours and depressive symptoms among Chinese workers using two-wave follow-up data from the China Family Panel Studies (CFPS). The findings provide valuable insights for evidence-based working time policies and mental health prevention and intervention strategies. First, we observed positive correlations between working hours and depressive symptoms, as well as significant positive associations between working hours and internet use. Results from the cross-lagged model indicated that working hours positively predicted depressive symptoms, internet use negatively predicted depressive symptoms, and working hours positively predicted internet use, suggesting complex interrelationships among these variables.

In contemporary society, work-related stress has progressively increased. Statistics indicate that the average working week for Chinese workers is approximately 48 h. Compared to European and North American countries, prolonged working hours in many Asian nations—particularly China, South Korea, and Japan—are detrimental to mental health (Virtanen et al., 2011) and significantly associated with suicide risk (Kong et al., 2023; Amagasa et al., 2005; Liu et al., 2024). Excessive working hours foster negative emotional states, thereby contributing to depressive symptoms (Virtanen et al., 2011).

Secondly, this study examined the mediating role of internet use in the relationship between working hours and depressive symptoms through a semi-longitudinal mediation model. Internet use demonstrated a significant mediation effect between prolonged working hours and depressive symptoms. Increased working hours may drive individuals to engage more frequently with the internet as a coping mechanism for work-related stress. Such internet use can partially alleviate depressive symptoms, reflecting a tendency to seek online entertainment or social interactions when confronted with occupational pressures (Liu et al., 2024). For example, Li et al. (2025a) found that diversified internet device use mitigated depressive symptoms in older adults by enhancing social engagement. Similarly, Qi et al. (2024) demonstrated that active participation in social media reduced the likelihood of developing depressive symptoms over 2 years. Nevertheless, despite these potential short-term benefits, sustained reliance on internet-based coping strategies may paradoxically exacerbate negative emotions over time.

A notable finding of this study is that internet use does not universally buffer the adverse effects of long working hours across all subgroups. Specifically, its mediating role in the relationship between working hours and depressive symptoms was statistically significant only among women and married individuals, particularly married female employees. This suggests that women, especially those who are married, may more actively seek emotional support online to alleviate depressive symptoms caused by occupational stress. Given the documented negative impact of prolonged working hours on the mental health of women and married workers (Qian and Sayer, 2016), our findings highlight the urgency of developing internet-based mental health interventions—such as online psychological counseling and supportive virtual communities (Han et al., 2018)—tailored to this demographic. Additionally, policymakers and employers should prioritize work–life balance initiatives for married women, including flexible scheduling and accessible mental health resources, as this group often faces compounded pressures from both domestic responsibilities and workplace demands. Prior research (Lee and Kang, 2023; Song et al., 2024) suggests that women are generally more psychologically vulnerable to mental health challenges, and marital role conflicts may exacerbate stress among working adults. Our findings corroborate these observations and underscore the important protective role of internet-mediated coping strategies for women and married workers experiencing work-related stressors.

Based on the urban–rural heterogeneity analysis, internet use showed a significant effect among rural workers, which can be understood in the context of China’s rapid digital development. Improved infrastructure has greatly increased rural residents’ access to online information, social interaction, and leisure, helping relieve work stress and enhance well-being—explaining the significant associations observed. However, the absence of a statistically significant mediation effect suggests that this mechanism is still in the process of emerging at the current stage of development. By contrast, urban populations, with earlier internet adoption and richer offline alternatives (e.g., cultural facilities, social venues), may rely less on the internet for stress management, which could explain the nonsignificant mediating role in this group.

However, age- and employment-specific analyses revealed significant protective effects only for younger and stably employed individuals, while the effects were nonsignificant among middle-aged adults, older adults, and those in precarious employment. In China, middle-aged workers—often burdened by multigenerational caregiving responsibilities as part of the “sandwich generation”—may struggle to achieve the anticipated emotional regulation through internet use. The dual economic and emotional demands of simultaneously supporting both children and aging parents often lead this group to use the internet in a more instrumental manner (e.g., searching for health information or educational resources) rather than for emotional regulation. This instrumental orientation may limit the internet’s potential to provide emotional support and compensation, thereby diminishing its mediating role in alleviating depressive symptoms. For precariously employed groups, structural constraints are even more evident: internet engagement cannot mitigate the fundamental financial insecurity or occupational uncertainty that underlie their elevated mental health risks. Prior research has consistently shown that workers in unstable employment face heightened vulnerability due to insufficient social safety nets (McKee-Ryan et al., 2005; Baek et al., 2024).

When internet use was further categorized by purpose, we found that online social interaction and entertainment were both effective in mitigating depressive symptoms, with entertainment showing a stronger protective effect. However, these benefits emerged only when the frequency of use reached at least once or twice per week. This finding offers practical implications for the design of internet-based clinical interventions, particularly those leveraging online entertainment platforms, to reduce the risk of depression.

Despite these valuable findings, this study has several limitations. First, the predominant reliance on self-reported measures for key variables—including working hours and depressive symptoms—may introduce biases such as recall inaccuracies and social desirability effects (Podsakoff et al., 2003). Second, constrained by data availability, our longitudinal mediation analysis was based on only two-wave panel data. To more fully capture the complex temporal dynamics and potential bidirectional effects among these variables, future studies should employ extended multi-wave cohort designs with finer temporal resolution. Finally, middle-aged individuals may encounter barriers in leveraging online social interaction as a source of emotional support, thereby limiting their opportunities to obtain positive affective compensation through internet use. Future research should examine the specific content, modes (e.g., active social engagement vs. passive content consumption), and habitual patterns of internet use in this population, in order to better understand how these factors shape their psychological benefits.

This study carries important policy implications. First, the finding that long working hours heighten depression risks highlights the need for stricter enforcement of labor regulations. Although China’s Labor Law limits work to ≤8 h/day and ≤40 h/week, with overtime capped at ≤36 h/month, 2024 data show workers averaging 48.6 weekly hours. The widespread “996 schedule” (9 a.m.–9 p.m., 6 days/week) reflects a persistent overtime culture that undermines rest. Second, the mental health benefits of social network use among women and married workers suggest the potential of psychosocial interventions via social media platforms. Workplace programs could integrate moderated online support groups tailored to these populations. Finally, for precariously employed workers, combining employment protections (e.g., universal basic labor contracts) with financial support could address underlying insecurities driving poor mental health. Moreover, appropriately regulating the frequency of internet use may enhance its effectiveness in alleviating depressive symptoms.

## 7 Conclusion

This study establishes a significant association between prolonged working hours and depressive symptoms among Chinese workers, with internet use serving as a partial mediating mechanism in this relationship. These findings provide preliminary evidence of the complex interplay between occupational time demands, digital behaviors, and mental health outcomes in China’s workforce. Future studies could employ more fine-grained occupational data or introduce occupation type as a moderating variable to explore whether significant heterogeneity exists in the relationship between internet use frequency and depressive symptoms across different occupational groups.

## Data Availability

Publicly available datasets were analyzed in this study. This data can be found at: the data that support the findings of this study are openly available in China Family Panel Studies at: https://www.isss.pku.edu.cn/cfps/index.htm.
